# Validation of hair ethyl glucuronide using transdermal monitoring and self‐reported alcohol use in women of childbearing potential

**DOI:** 10.1002/npr2.12151

**Published:** 2021-03-02

**Authors:** Andrea L. Blair, Ashleigh L. Chiaf, Erica K. Crockett, Tracy Kent Teague, Julie M. Croff

**Affiliations:** ^1^ Oklahoma State University Center for Health Sciences National Center for Wellness & Recovery Tulsa OK USA; ^2^ Integrative Immunology Lab Department of Surgery University of Oklahoma‐Tulsa Tulsa OK USA; ^3^ Department of Rural Health Oklahoma State University Center for Health Sciences Tulsa OK USA

**Keywords:** alcohol, biosensor, ethyl glucuronide, hair EtG, transdermal

## Abstract

**Aim:**

The present study aimed to evaluate the validity of hair ethyl glucuronide concentrations compared with transdermal alcohol concentration and self‐reported alcohol use.

**Methods:**

This trial included 25 adolescent and young adult females, aged 16‐24, who reported at least one heavy drinking episode (≥4 drinks) in the two weeks prior to baseline. All participants were asked to wear an alcohol biosensor over a one‐month prospective study. Detailed self‐report of drinking behaviors was assessed weekly. Estimates of blood alcohol concentration were computed from self‐report data using the National Highway and Transportation Safety Administration equation. Transdermal alcohol concentration and estimated blood alcohol concentration data were categorized into at‐risk (>0.05 g/dL alcohol) and high‐risk (>0.08 g/dL alcohol) drinking events. Hair ethyl glucuronide concentration, total number of drinking events, moderate (>0.05 g/dL) and high level (>0.08 g/dL) of transdermal alcohol concentration, and estimated blood alcohol concentration drinking events were analyzed with Spearman's rank correlation test for validity comparisons.

**Results:**

No significant correlations were found between hair ethyl glucuronide values and total number, and moderate or high levels of detected drinking events by estimated blood alcohol concentration or transdermal alcohol concentration. Total number of drinking events detected and number of drinking events >0.08 g/dL using estimated blood alcohol concentration and transdermal alcohol concentration methods were significantly correlated with each other (respectively, *R* = .33, *P* < .05; *R* = .42, *P* < .05).

**Conclusion:**

Our findings indicate that, due to the number of false negatives, hair ethyl glucuronide concentrations should be used with caution for monitoring abstinence from alcohol use.

## 1 INTRODUCTION

Alcohol is the leading global lifestyle cause of death among 15‐ to 49‐year‐olds,^1^ contributing to one in twenty deaths globally in 2016.^2^ Alcohol use is particularly concerning among adolescents and young adults, who represent a significant economic investment to society and who suffer disproportionately from the consequences of alcohol use.^3^ More than 4000 adolescents and young adults (under the age of 21) die each year in the United States from alcohol‐related causes.^4^ Many US adolescents engage in risky alcohol use, despite multiyear trends of decline. Nationally, in the past 30 days, nearly one in three (30%) reported drinking alcohol and nearly one in five (19%) reported engaging in heavy episodic drinking (4+ drinks for females or 5+ drinks for males in one setting).^3^

Biological sex is also a critical consideration of risk associated with alcohol use. Rates of use have historically been highest among males; however, the gap in alcohol use between sexes is waning, particularly among young adults.^5^ This trend is largely attributable to societal gender equality.^6^ In the United States, females (62.6%) are more likely to engage in underage drinking than males (58.1%).^3^ Rates of heavy and binge drinking among US women have increased by more than 50% over the past decade,^7^ with peak episodic consumption occurring during childbearing years (age 18‐44).^5^ The childbearing potential of underage and young adult female drinkers further amplifies the risks among this age and gender group because these behaviors continue into early pregnancy and pregnancy is known later among women who drink.^8^ Accurate measurement of intoxication from alcohol, particularly in early stages of use, is critically important to better understand the potential for intergenerational risk associated with unintended and alcohol‐exposed pregnancy.

Alcohol use behaviors can be monitored by self‐report, direct measurement of recent drinking events, or through specialized biological sampling. Each methodology may allow examination for specific windows of use. Participant self‐reported use is a standard in the field and allows examination across multiple time frames. Direct measurement of alcohol using blood, breath, or transdermal approaches can provide data on a current or very recent drinking event, due to the rapid metabolism of alcohol in the body. Emerging techniques in the field include sampling of metabolites of alcohol in hair or nails which can provide a retrospective assessment of consumption within a several month time frame.^9^, ^10^

Participant self‐reports for measurement of alcohol use have been used for research and in clinical settings.^11^ Self‐reported alcohol use can be used to calculate estimated blood alcohol concentration (eBAC) when in vivo blood alcohol concentrations are inconvenient or unattainable.^12^ There is some controversy associated with the use of eBAC compared with direct measurements of alcohol intoxication; however, eBAC has been shown to be highly correlated with actual blood alcohol concentration.^13^ Self‐report of drinking events and transdermal alcohol sensor data are also highly correlated.^14^, ^15^, ^16^ When participants are asked to recall drinking events after an extended period of time, self‐reports underestimate drinking^17^; however, newer methods using shorter recall time spans have increased the accuracy and decreased recall bias.^18^, ^19^

The Wrist Transdermal Alcohol Sensor (WrisTAS) is an electrochemical sensor device resembling a wristwatch.^20^ Ethanol detected by transdermal devices generally reflects blood alcohol levels, BAC.^21^ These devices are often used for monitoring individuals in substance abuse treatment programs and intervention research among adult populations.^15^, ^22^, ^23^, ^24^, ^25^ Transdermal alcohol sensors can be used to approximate, but not directly derive, blood alcohol concentration and may be inconsistent in detection of low‐intensity drinking events.^14^, ^16^, ^26^, ^27^

Emerging techniques allow for the indirect measurement of previous drinking events through direct metabolites including ethyl glucuronide (EtG)^28^ and ethyl sulfate (EtS). EtG is a minor metabolite of alcohol and can be measured in urine,^29^ hair,^30^ and nails.^31^ EtG accumulates in the hair shaft through perspiration.^30^, ^32^, ^33^, ^34^, ^35^ Currently, hair sampling is employed to monitor alcohol use via EtG in alcohol and drug treatment programs.^35^ Hair EtG has been shown in a number of studies to be a reliable measure of chronic alcohol use.^30^, ^32^, ^33^, ^36^, ^37^, ^38^, ^39^ Studies have shown that hair EtG has an extremely low rate of false positives; therefore, a positive result is strongly associated with chronic consumption of alcohol.^30^, ^37^, ^40^ Results of studies have shown the impact of cosmetic treatments,^10^ hair color,^41^ gender,^10^, ^28^, ^42^, ^43^ and level of drinking, such as heavy episodic drinking, occasional use, or chronic use.^38^, ^39^, ^42^, ^44^ However, the 2019 consensus from the Society of Hair Testing (SoHT) states that natural hair color and ethanol‐containing hair care products do not influence EtG concentrations,^45^ nor does hair washing.^46^

Despite several studies which have examined validity of hair EtG in chronic heavy drinkers^32^, ^34^, ^36^, ^47^ and factors, such as hair color, that may affect EtG values,^37^, ^38^, ^39^, ^48^ there have been no studies to date that have looked at hair EtG as a method of monitoring alcohol use in adolescent and young adults with intermittent heavy drinking events. The purpose of this manuscript was to evaluate and identify valid measures of alcohol consumption among adolescent and young adult women. This manuscript presents a comparison of hair EtG, transdermal alcohol concentration (TAC), and self‐reported alcohol (eBAC) use in participants, with inconsistent levels of alcohol consumption.

## 2 METHODS

### 2.1 Participants and criteria

Participants were recruited as part of a larger study examining alcohol effects on nutriture. Recruitment of participants largely occurred through respondent‐driven sampling. Potential participants were directed to the study website for initial screening. The study website contains a video explaining the study, frequently asked questions, and a link to a screening survey.

Individuals qualified for the study if they met the following criteria: female, between the ages of 14 and 24, live within a 45‐minute radius of the research office, and reported at least one heavy drinking episode (≥4 standard alcoholic drinks) on one occasion within the past 2 weeks. Only female participants were recruited as this is part of a larger trial examining periconceptional behaviors in women of childbearing potential. When potential participants met the screening criteria, a member of the research team called to rescreen the potential participant and schedule a consent and baseline appointment. Parents were required to accompany any potential participant under 18 years of age; her parents were required to consent and she to assent to her participation in the study. Anthropometric data collected at baseline included height, weight, and waist circumference. Self‐reports were collected from participants on a weekly basis; self‐report included day of drinking events, amounts and types of alcohol use, and length of drinking events. All participants were asked to wear a Giner WrisTAS sensor over a one‐month prospective study. In a subsample of the study population (n = 25), a hair sample was taken at the end of the four‐week longitudinal study and sent for analysis. This subsample was based on participants who had at least one heavy drinking episode within the past 14 days (≥4 standard alcoholic drinks) and ability to obtain a sufficient quantity of hair sample. The National Highway and Transportation Safety Administration (NHTSA) equation was used to compute eBAC from self‐report data.

### 2.2 Transdermal alcohol sensor (TAS)

The Giner WrisTAS generation 7 (21 participants) and generation 9 (4 participants) were utilized to detect ethanol vapor and as a data acquisition–recording device. This device consists of three components: a sensor to detect ethanol vapor from the skin, thermistors which produce temperature signals, and a data‐recording device to store data.^14^ Participants wore the WrisTAS sensors for at least 21 hours per day for the study period of 28 days. Each device continuously sampled ethanol vapor detected near the skin, as well as temperature, humidity, and skin conductivity at 5‐minute intervals. Members of the research staff informed the participants how to adjust the straps on the sensor and to remove the sensor while exercising or showering to prevent water exposure. Both WrisTAS generation 7 and generation 9 are water resistant, but not waterproof. Participants were instructed to wear the sensor on their ankle. While these sensors are marketed to be worn on the wrist the manufacturer's website states “you can wear it discretely on your wrist, or on just about any part of your body where blood vessels are close to the skin surface”.^49^ The participants were instructed they could move the sensor from one ankle to another depending on comfort. Finally, participants were instructed to avoid placing lotion or perfumes on the ankle on which they wore the sensor. Data were downloaded from the sensor weekly throughout the month‐long study. The data are stored in the sensor unit until connected via a serial port to a computer for download. Sensors were maintained weekly, including battery changes in both generation 7 and generation 9, and addition of deionized water to the generation 7 sensors. The timing of maintenance, and subsequent quality control, resulted in the participants being fitted with a new monitor each week. Sensor data are reported as transdermal alcohol concentration (TAC), with values that correlate with blood alcohol concentration (BAC).

### 2.3 Estimated blood alcohol concentrations (eBACs)

Blood alcohol concentration was estimated using the National Highway Traffic Safety Administration (NHTSA) equation with data collected as part of the study. Weights of participants were measured during the baseline visit. Each week, participants completed a retrospective report of alcohol use following a timeline follow‐back (TLFB) approach. The research staff asked participants to report on which days they drank alcohol. Additionally, participants were asked to report how long each drinking event lasted as well as the number of standard drinks of beer, wine, liquor/spirits, and mixed drinks consumed (the following standard drink equivalents were used: 12‐ounce beer or wine cooler, 5‐ounce glass of wine, and 1.5 ounce of liquor). Participants were grouped into total number of drinking events, total number of moderate and binge drinking events >0.05 g/dL, and total number of binge drinking events >0.08 g/dL.

### 2.4 Hair ethyl glucuronide (EtG)

Hair samples were collected from each participant by cutting the hair with scissors close to the scalp near the posterior vertex (crown) region of the head. The proximal end of each sample was marked. The samples were analyzed for EtG at US Drug Test Centers Analysis. All analyses were conducted according to validated and published methods.^50^, ^51^ Each hair sample was trimmed, and only the proximal 0.5 inch was analyzed for EtG concentrations. Hair samples were washed to remove possible external contamination of EtG, dried, and then pulverized. Pulverizing, or powdering, the hair increases the surface area from which EtG can be extracted,^10^ and has been shown to significantly increase EtG concentration compared with cutting the hair into small pieces.^52^, ^53^ Liquid chromatography coupled with tandem mass spectrometry (LC/MS/MS) was used to analyze EtG in hair samples. The limit of detection/quantification (LOD/LOQ) for EtG was 2 pg/mg, as is recommended by SoHT.^45^

### 2.5 Risk categorization

Participants completed self‐report questionnaires on frequency and quantity of alcohol consumption as well as any episodes of binge drinking. Participants were classified into four categories (teetotalers, low‐risk drinkers, at‐risk drinkers, and high‐risk drinkers) based on average grams per day of alcohol consumed. Guidelines for categorization of consumption were based on WHO recommendations and current literature. Teetotalers were defined as participants who declared total abstinence from alcohol consumption (0 g alcohol/day) during the 30‐day trial. Low‐risk drinkers were defined as participants who consumed ≤20 g alcohol/day (equivalent to <1 standard alcoholic drink per day). At‐risk drinkers were defined as participants who consumed >20 g alcohol/day but <60 g alcohol/day (equivalent to 1‐2 standard alcoholic drinks per day). High‐risk drinkers were defined as participants who consumed >60 g alcohol/day (equivalent to >2 standard alcoholic drinks per day). Ethyl glucuronide concentrations in hair samples were utilized to assign participants into three categories. Low‐risk drinkers were defined as participants with EtG concentration of ≤5 pg/mg; this category was set at the limit that does not contradict self‐reported alcohol abstinence based on new 2019 Society of Hair Testing (SoHT) recommendations.^45^ At‐risk drinkers were defined as those participants with EtG concentration >5 pg/mg and <30 pg/mg. High‐risk drinkers were defined as participants with EtG concentration ≥30 pg/mg.^45^, ^48^

### 2.6 Statistical methods

Statistical evaluations were carried out using JMP© version Pro 14 software (SAS Institute Inc) and IBM SPSS Statistics 26 (IBM Corp.). Correlation coefficient (*R*) between EtG values, TAC groups, and eBAC groups were calculated. The Pearson correlation test was used for Gaussian distribution cases whereas the nonparametric Spearman rank correlation test was applied for non‐Gaussian distribution cases. Chi‐square analysis was used to determine whether there was an association between categorical variables of race, ethnicity, age groups (≤20 and ≥21), BMI groups (normal 18.5‐24.9, overweight 25‐29.9, obese 30‐39.9, extremely obese >40), education level of mother (as a descriptor of socioeconomic status),^54^ and employment status of the participant. Sensitivity (true positive/total positives), specificity (true negative/total negative), positive predictive value (PPV) (% of positives that are positive), and negative predictive value (NPV) (% of negatives that are negative) of hair EtG were calculated and compared for detection of alcohol consumption in different subgroups.

## 3 RESULTS

Of the 25 participants, the data included 700 days of continuous alcohol monitoring. Demographics for study population and comparison of alcohol consumption among risk groups are presented in Table 1. The mean age of participants was 19.88 years (±SD 2.74), and mean BMI was 26.07 (±7.15). Based on self‐reported alcohol use, participants fell into two risk groups: low‐risk drinkers, ≤20 g alcohol/day (n = 18, 72%), and at‐risk drinkers, >20 g alcohol/day (n = 7, 28%). Hair EtG results indicated individuals in every risk stratum: low‐risk drinkers (n = 21, 84%), at‐risk drinkers (n = 2, 8%), and high‐risk drinkers (n = 2, 8%). There were no significant differences in race, ethnicity, BMI, mother's education level, or employment status across EtG and self‐report risk groups (Table 1). Significant differences by alcohol use risk were found by age groups based on results from self‐report, and younger participants (<20 years of age) were less likely to self‐report at‐risk consumption than older participants (*P* < .001). Based upon the risk groupings for hair EtG, this could indicate that younger participants under‐report alcohol consumption and older participants may over‐report alcohol use.

**TABLE 1:** Demographic characteristic and comparison of hair EtG concentration (pg/mg) and eBAC (g/dL) in each alcohol consumption subgroup

	Demographic characteristics	EtG risk groups				Self‐report risk groups		
		Low‐risk drinkers (n = 21)	At‐risk drinkers (n = 2)	High‐risk drinkers (n = 2)	*P*‐value	Low‐risk drinkers (n = 18)	At‐risk drinkers (n = 7)	*P*‐value
Race								
Caucasian, n (%)	18 (72)	15 (83)	1 (6)	2 (11)	.604	13 (72)	5 (28)	.970
American Indian/Alaskan Native, n (%)	3 (12)	3 (100)	0	0		2 (67)	1 (33)	
Other, n (%)	4 (16)	3 (75)	1 (25)	0		3 (75)	1 (25)	
Ethnicity								
Hispanic/Latino, n (%)	4 (16)	3 (75)	1 (25)	0	.342	3 (75)	1 (25)	.884
Non‐Hispanic/Latino, n (%)	21 (84)	18 (86)	1 (5)	2 (9)		15 (71)	6 (29)	
Age								
< 21, n (%)	14 (56)	12 (86)	1 (7)	1 (7)	.966	14 (100)	0	<.001
≥ 21, n (%)	11 (44)	9 (82)	1 (9)	1 (9)		4 (36)	7 (64)	
BMI								
Normal, n (%)	14 (56)	13 (93)	0	1 (7)	.430	12 (86)	2 (14)	.104
Overweight, n (%)	6 (24)	5 (83)	1 (17)	0		4 (67)	2 (33)	
Obese, n (%)	4 (16)	2 (50)	1 (25)	1 (25)		1 (25)	3 (75)	
Extremely Obese, n (%)	1 (4)	1 (100)	0	0		1 (100)	0	
Mother's education								
Less than high school, n (%)	1 (4)	1 (100)	0	0	.712	1 (100)	0	.452
High school or GED, n (%)	5 (20)	3 (60)	1 (20)	1 (20)		3 (60)	2 (40)	
Some college, n (%)	6 (24)	6 (100)	0	0		5 (83)	1 (17)	
College graduate, n (%)	7 (28)	7 (100)	0	0		6 (86)	1 (14)	
Graduate or professional degree, n (%)	5 (20)	3 (60)	1 (20)	1 (20)		2 (40)	3 (60)	
Employment								
Employed, n (%)	19 (76)	17 (90)	1 (5)	1 (5)	.414	12 (63)	7 (37)	.080
Not employed, n (%)	6 (24)	4 (66)	1 (17)	1 (17)		6 (100)	0	

The total number of drinking events detected using eBAC was significantly correlated with the total number of drinking events detected using TAC methods (*R* = .33, *P* < .05). The number of drinking events >0.08 g/dL detected using eBAC was significantly correlated with the number of drinking events >0.08 g/dL detected using TAC methods (*R* = .42, *P* < .05). However, no statistically significant correlations were found between hair EtG values and the number of self‐reported drinking events by eBAC or detected drinking events by TAC (Table 2). Further, no significant correlation was identified between the number of events with eBAC from low‐risk (>0.05 g/dL) and at‐risk (>0.08 g/dL) drinking, and the number of events with TAC from low‐risk (>0.05 g/dL) and at‐risk (>0.08 g/dL) and hair EtG values (Table 2).

**TABLE 2:** Correlations for main variables

	1	2	3	4	5	6	7
1. Hair EtG value	—						
2. Total number of self‐reported drinking events	‐.0406	—					
3. Number of self‐reported drinking events where eBAC >0.05 g/dL	.0178	.8144***	—				
4. Number of self‐reported drinking events where eBAC >0.08 g/dL	.1600	.5107**	.7933***	—			
5. Total number of sensor‐detected drinking events	‐.0771	.3282*	.3093	.3403	—		
6. Total number of sensor‐detected drinking events where TAC >0.05 g/dL	‐.0798	.3362	.2935	.3844	.8170***	—	
7. Total number of sensor‐detected drinking events where TAC >0.08 g/dL	‐.0005	.1976	.2279	.4239^*^	.7062***	.8987***	—

Note

All drinking events were recorded over a 28‐day period.

^*^P < .05, **P < .01, ***P < .001 two‐tailed. N = 25. Nonparametric Spearman's rank correlation test.

Sensitivity, specificity, positive predictive values, and negative predictive values of hair EtG for detecting alcohol consumption are displayed in Table 3. Sensitivity of hair EtG was consistently low (0.29‐0.35), while specificity was high in most cases (0.75‐1). The true positive rate was negatively affected by the number of false negatives. In all cases, if hair EtG was positive we found that the participant's self‐report also showed that the participant drank; therefore, a positive EtG sample equaled a drinking event.

**TABLE 3:** Sensitivity, specificity, positive predictive value (PPV), and negative predictive value (NPV) of EtG to detect alcohol consumption

Hair EtG vs	Sensitivity	Specificity	PPV	NPV
Total number of self‐reported drinking events	0.32	0	1	1
Total number of self‐reported drinking events where eBAC >0.05 g/dL	0.33	1	1	0.94
Total number of self‐reported drinking events where eBAC >0.08 g/dL	0.35	1	1	0.88
Total number of sensor‐detected drinking events	0.33	1	1	0.94
Total number of sensor‐detected drinking events where TAC >0.05 g/dL	0.29	0.75	0.75	0.88
Total number of sensor‐detected drinking events where TAC >0.08 g/dL	0.32	0.75	0.75	0.77

## 4 DISCUSSION

This study found hair EtG tests to be a very low sensitivity assessment. Total self‐reported drinking events and TAC‐detected drinking events were moderately and significantly correlated; however, hair EtG did not correlate to these other alcohol detection methods. Hair EtG testing among women has the ability to indicate alcohol exposure during pregnancy, with sampling taken in the second trimester indicative of first‐trimester exposures. Hair EtG could potentially impact both clinical and legal decisions and is increasingly being implemented as standard practice in courts and treatment programs as a tool for ensuring participant compliance^40^; moreover, among women tested during pregnancy, this may impact rates of child removal from the home. Hair EtG has previously been used for confirmatory testing of alcohol use or abstinence among heavy alcohol–using^30^, ^32^, ^33^, ^36^, ^37^, ^38^, ^39^ and light alcohol–using participants.^42^, ^43^ In this study of women of childbearing potential, hair testing identified only 8% of at‐risk drinkers (>5 pg/mg), and 8% of high‐risk drinkers (>30 pg/mg), with no correlation between hair EtG and level of consumption detected via other methods. The high rate of abstinence recorded (< level of detection (LOD), n = 16, ≤5 pg/mg, n = 5, total n = 21, 84%) through hair EtG raises serious concerns about the ability of these tests to confirm abstinence in a population of intermittent alcohol consumers. Results of our study add to the findings of other studies that a negative result, or less than level of detection (LOD), does not necessarily exclude alcohol consumption, especially if consumption levels are inconsistent.^30^, ^40^, ^55^

In this study, the population represented female individuals with varying levels of alcohol consumption. Participants consumed, on average, anywhere from <1 standard drink (14 g) to more than 24 (336 g) standard drinks per week. Sensitivity analysis, the true positive rate, revealed that eBAC number of drinking events ≥0.08 g/dL and eBAC number of drinking events ≥0.05 g/dL provided only weak sensitivity values (0.35 and 0.33, respectively) which are approximately a one in three chance of a positive EtG detection. Detection rate, especially in the heavy drinking group, should be closer to 1.0 to be clinically useful. The PPV of 1.00 did indicate with certainty that the positive samples were indeed true positive values.

Despite the strengths of the design, there are several potential limitations of this study. First, this study cannot assess the role of EtG in confirming abstinence from alcohol, due to all participants self‐reporting alcohol consumption and had biosensor‐detected drinking events. Second, the interpretation of a hair EtG result is sensitive to the use of hair products to highlight or bleach hair, causing a reduction in the level of EtG detectable in the hair shaft.^40^ Chemical and/or thermal hair treatments were not recorded in this study. Third, Huestis and colleagues found that in the portion of hair cut for a typical sample, representation of drinking behavior in the past week is unrealistic as alcohol metabolites take at least a week to enter the hair shaft.^56^ Samples were taken on day 28 of study and therefore may have captured data from 7 days prior to baseline through day 21 of the study.

In accordance with other studies, EtG was not consistently identified even in our highest level drinking groups. Other studies have found variability in the ability to detect EtG levels above SoHT guidelines in a controlled study with known consistent heavy drinkers and social drinkers.^55^ Our findings indicate that, due to an unacceptably high number of false negatives, current EtG analysis techniques have difficulty consistently monitoring alcohol abstinence or consumption and should be used with caution. Future studies should include a longer study time, recording of chemical or thermal hair treatments, and a more diverse population; these results are limited by an underrepresentation of African American and Asian participants. Moreover, additional research should include to what extent, if any, texture of hair affects EtG concentrations.

## CONFLICT OF INTEREST

The authors declare no conflict of interest.

## AUTHOR CONTRIBUTIONS

AB, AC, EC, KT, and JC have made substantial contribution to the conception, study design, data collection, data analysis, and/or interpretation of data and a contribution to manuscript writing and intellectual content of the article; and acknowledge that they have exercised due care in ensuring the integrity of the work.

## APPROVAL OF THE RESEARCH PROTOCOL BY AN INSTITUTIONAL REVIEWER BOARD

The protocol for this research project has been approved by the Oklahoma State University Institutional Review Board, and a certificate of confidentiality was obtained from NICHD.

## INFORMED CONSENT

Written informed consent was obtained from all participants and/or guardian(s) after a complete explanation of the procedures by researchers at the study facility. Participants were able to refuse study participation.

## REGISTRY AND THE REGISTRATION NUMBER OF THE STUDY/TRIAL

N/A.

## ANIMAL STUDIES

N/A.

## Data Availability

The data that support the findings of this study are available on request from the corresponding author. The data are not publicly available due to privacy restrictions.
